# Development and Validation of a Food and Nutrition Literacy Questionnaire for Chinese Adults

**DOI:** 10.3390/nu14091933

**Published:** 2022-05-05

**Authors:** Yaqin Zhang, Zhaofeng Zhang, Meihong Xu, Sumiya Aihemaitijiang, Chen Ye, Wenli Zhu, Guansheng Ma

**Affiliations:** Department of Nutrition & Food Hygiene, School of Public Health, Peking University Health Science Center, Beijing 100191, China; zyqbjdx@163.com (Y.Z.); zhangzhaofeng@bjmu.edu.cn (Z.Z.); 1410606101@pku.edu.cn (S.A.); 1510306235@pku.edu.cn (C.Y.); zhuwenli@bjmu.edu.cn (W.Z.); mags@bjmu.edu.cn (G.M.)

**Keywords:** food and nutrition literacy, questionnaire development, Chinese adults, validation

## Abstract

The purpose of this study aimed to develop and validate the Food and Nutrition Literacy Questionnaire for Chinese adults (FNLQ). The dimensions and core components of Food and Nutrition Literacy were constructed though literature review and qualitative consensus study. A cross-sectional survey of 8510 participants was conducted. The reliability of the questionnaire was determined by internal consistency, the construct validity was assessed by exploratory factor analysis (EFA) and confirmatory factor analysis (CFA), and the content validity was assessed by the Pearson correlation coefficient. From the literature review and qualitative methods, 20 core components and 50 questions of the FNLQ were developed, including 1 dimension of knowledge and 3 practice dimensions (ability of selection, preparing food and eating). The overall FNLQ questionnaire had good reliability and validity (Cronbach’s α = 0.893, χ2/DF = 4.750, RMSEA = 0.048, GFI = 0.891 and AGFI = 0.876). The average FNLQ score of all participants was (64.08 ± 12.77), and the score for the knowledge and understanding dimension was higher than that for the practice dimensions. In addition, 80 was set as the nutritional literacy threshold, and only 12.2% met this threshold in this survey. Sociodemographic and health status characteristics were predictors of FNLQ (R^2^ = 0.287, F = 244.132, *p* < 0.01). In conclusion, the FNLQ built in this study had good validity and reliability. It could be considered as a reliable tool to assess Food and Nutrition Literacy of Chinese adults.

## 1. Introduction

It is well known that dietary risks were responsible for 22% and 15% all death and all disability-adjusted life years (DALYs), respectively, worldwide [1]. The numbers of the China part were much higher, which were 30.2% and 21.3%, respectively. According to the 2021 Global Nutrition Report, nearly one-fifth of the world’s diseases are related to malnutrition, mainly in the form of nutrition inadequate intake coexisted with nutritional imbalance. Additionally, the rapid growth of chronic diseases was associated with malnutrition. The imbalance of nutrition (means the double burden of malnutritional and overnutrition) were trigged by poor dietary patterns and exacerbated the occurrence and development of chronic non-communicable diseases [2]. In the past few decades, the spectrum of disease and death have been changed dramatically in China [3,4]. Low weight rate was declined (4.2% vs. 6.0%, 2015 vs. 2020), but overweight and obesity rates were increased (34.3% vs. 30.1% and 16.4% vs. 11.8%, respectively, 2015 vs. 2020). Obesity and overweight are becoming more prominent, and the prevalence and incidence of major chronic diseases are on the rise. Unhealthy lifestyles are still prevalent. The daily dietary energy ratio (per standard person) and salt intake were 34.6% and 9.3 g higher, respectively, than the upper limit of recommendations. On the contrary, low intake of fruits and vegetables, lower than the 50% of the recommendation, was present 30.2% and 87.1%. It has been pointed that out one of keys is changing the dietary structure of Chinese residents [5,6].

Nowadays, the relationship, between nutrition literacy, dietary behavior, and nutritional and even health status has been revealed. It is worth noting that both nutrition literacy and dietary behavior can be improved by nutrition education and management [7]. As the upstream of dietary behavior, nutrition literacy is the most important factor affecting dietary intake. Thus, improving nutrition literacy has been regarded as an effective strategy to promote nutrition status and health. The improvement of its level is the most fundamental, economical, and effective measure to promote nutritional health and is of great value in national health promotion [8,9,10,11]. The nutritional health level of the general population is an important dimension to evaluate the level of national human resources.

People could choose healthier food if they had sufficient nutrition literacy [12]. Nutrition literacy was defined as an individual’s ability to obtain, process, and understand basic nutrition information and use these abilities [13,14,15], including the ability to interpret nutrition policies, read nutrition labels, and choose healthier foods from different foods. It plays a crucial role in the prevention and control of nutrition-related diseases [14,15,16,17,18,19]. There are several instruments for measuring food and nutrition literacy, such as the Food and Nutrition Literacy Questionnaire (FNLIT) [15], the Nutrition Literacy Assessment Instrument (NLAI) [20], the Nutrition Literacy Scale (NLS) [21], the Critical Nutrition Literacy Scale (CNL) [22], and the Newest Vital Sign (NVS) [23]. Considering the dietary culture gaps among different countries, the above instruments cannot be used for assessing Chinese adults. Although health literacy has been monitored nationwide since 2008 in China, there are only 10 items (10/66) related to nutrition and eating habits in the Chinese Citizens’ Health Literacy guide (2015), and most of these items focus on the knowledge dimension. Nutrition literacy is based on health literacy, but more than that, the literacy, especially functional literacy, is situation specific; that is why there is distinction between nutrition and health literacy. However, there are no food and nutrition literacy assessment instruments specifically developed and validated for Chinese adults.

Thus, the present study aimed to develop and validate the Food and Nutrition Literacy Questionnaire (FNLQ) for Chinese adults to provide an effective tool for assessing and monitoring the level of nutrition literacy and nutrition education of Chinese residents.

## 2. Materials and Methods

**Figure 1:**
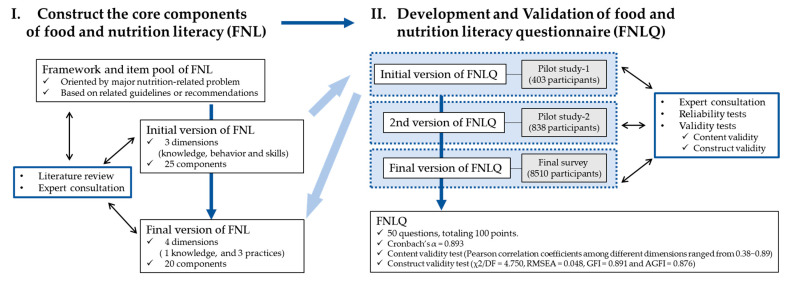
Study flow.

In this study, the process of development and validation is shown in the Figure 1.

### 2.1. Development of Questionnaire

The framework of Food and Nutrition Literacy is defined in line with a previous study by our team [6]. The development of FNLQ mainly comprised two stages:

Stage 1: construct the core components of Food and Nutrition Literacy

Literature review and qualitative consensus study were conducted to build the conceptual framework and dimensions of Food and Nutrition Literacy. A face-to-face expert panel meeting were held to discuss and preliminary determinate the framework in September 2019. The experts were qualified with adequate experience in nutrition, food, and health education, who would participate in the following content validity index method, in which their detailed information would be presented.

Then, a qualitative consensus study was conducted to determine the dimensions and core components of the FNLQ, a two-round electronically distributed content validity index test. Each item of the questionnaire was assessed by 12 experts. The content validity index study was conducted from September 2019 to December 2019. A questionnaire with the outline of provisional Food and Nutrition Literacy core components was mailed to each expert in the first-round survey. The scoring was conducted quantitatively, where the experts determined its relevance using the following scale: (1) not relevant, (2) quite relevant, (3) relevant, and (4) highly relevant. Scores of (1) and (2) were deemed irrelevant and given a value of 0, while scores of (3) and (4) were deemed relevant and given a value of 1. Furthermore, the calculation was based on the Item-Level Content Validity Index (I-CVI) and Scale-Level Content Validity Index (S-CVI). The I-CVI and S-CVI were used to demonstrate validity. S-CVI/Ave, the mean of I-CVI value for each item of the questionnaire, was also used. Content Validity Indices were considered acceptable when I-CVI > 0.78 and S-CVI > 0.90, respectively [21]. After the discussion of the summary of first-round survey, the components of Food and Nutrition Literacy were revised. Finally, the second-round survey was implemented until a compromise was reached.

Stage 2: Develop the FNLQ

According to the core components, a pool of 60 questions was generated to measure the core components of Food and Nutrition Literacy. The questionnaire consisted of two parts: the first part investigated sociodemographic characteristics (age, gender, and education levels) and health status by a self-report questionnaire; the second part was the Chinese Food and Nutrition Literacy assessment scale. The questions included 5-point Likert-type questions (“Good dietary patterns are the foundation of adequate nutrition: strongly disagree, disagree, do not know, agree, strongly agree,”), choice questions (“What is the approximate weight of a ping-pong-ball-sized egg?”). The appropriateness of the questionnaire was evaluated by food and nutrition experts in the study steering group. After redundant components were eliminated, the final questionnaire included 50 questions, totaling 100 points. The higher the score, the higher the Food and Nutrition Literacy level of the respondents.

### 2.2. Validation of Questionnaire

#### 2.2.1. Data Collection

The participants were voluntarily recruited from April to July 2021. The investigators explained the investigation protocol to all 10,000 participants. Finally, e-written informed consent was obtained from 8510 participants, and the response rate was 85.10%. five to ten times the number of questions were considered a rational amount to perform the reliability and validity tests. The FNLQ contains 50 questions, so the sample size was between 250 and 500. Approximately 10% of the subjects were randomly selected as the sample size for the reliability analysis [22].

#### 2.2.2. Reliability Tests

The internal consistency reliability was measured by calculating the Cronbach’s alpha coefficient of the overall questionnaire as well as of each dimension and each component. For the overall questionnaire, a coefficient greater than 0.7 indicated acceptable reliability [23,24,25]. 

#### 2.2.3. Validity Tests

Exploratory factor analysis (EFA) and confirmatory factor analysis (CFA) were used to assess the construct validity of the FNLQ scale. The Kaiser–Meyer–Olkin (KMO) measure (≥0.6) was used to determine the sampling adequacy. Bartlett’s test of sphericity (*p* < 0.05) and total variance explained were used for EFA. Then, principal component analysis and the maximum variance method were used to explore the factorial pattern (determined by the number of common factors, load value, variance of common factors and variance contribution rate of factors). For the CFA part, root mean square error of approximation (RMSEA) equal to or smaller than 0.08 was considered an acceptable fit (≤0.05 as a good fit). The goodness-of-fit index (GFI) and adjusted goodness-of-fit index (AGFI) were selected as incremental fit indices. When the values of GFI and AGFI were at or above 0.85, they were considered acceptable. In this study, only the practice domain was analyzed by EFA and CFA. 

The content validity of components, dimensions, and the overall questionnaire was assessed by the Pearson correlation coefficients. A coefficient greater than 0.6 suggested that the components and dimensions had good discrimination and correlation with the overall index.

### 2.3. Statistical Analysis

All the data should be reviewed by the investigator. After excluding the unqualified questionnaires, all questionnaires were entered through EpiData. Internal consistency and other parametric tests were computed by using SPSS and AMOS 24.0(SPSS, Inc., Chicago, IL, USA). The final score was converted into a centesimal measure for comparison. Multiple and Logistic linear regression analysis was used to explore the related factors of Food and Nutrition Literacy. The significance level was set at *p* < 0.05.

## 3. Results

### 3.1. Core Components of Food and Nutrition Literacy

Two-round content validity index consultation was conducted. The response rates of both rounds were 100%, and the average authority degree of the experts was 0.923. Finally, 20 core components of the FNLQ were determined, including 1 dimension of knowledge; 3 practice dimensions (ability of access and selecting, preparing, and eating); and 3 levels of functional, interactive, and critical literacy. The I-CVI and S-CVI were calculated as shown in Table 1.

Based on the validity test, the study produced I-CVI scores ranging from 0.83 to 1.0, with S-CVI scores of 0.98, 0.93, 1.00, and 0.93 on the food and nutrition knowledge and understanding, ability of access and selecting food, preparing food, and healthy eating scales, respectively. The results show that the components were deemed relevant in measuring the knowledge, and practice of an individual regarding food nutrition literacy.

### 3.2. Demographic Characteristics of Participants

A total of 8510 participants tolled in the study, including 4578 male (53.8%) and 3933 female (46.2%). Among those, samples (*n* = 841) were used to analyze the reliability and validity of the questionnaire, and the total samples (*n* = 8510) were used for the final study. The sociodemographic and health status characteristics of the two study samples are shown in Table 2. According to the BMI cutoff in dietary guidelines for Chinese residents (2016) [26], there were 5810/586 (68.3/69.7%) and 2700/255 (31.7/30.3%) participants with appropriate (18.5~23.9) and abnormal (<18.5 or ≥24.0) BMI, respectively. 

### 3.3. Reliability

The Cronbach’s α coefficients for overall and the four dimensions (knowledge, selecting food, preparing food, eating) were 0.893, 0.866, 0.845, 0.812, and 0.816, respectively. An additional α test that deleted components one at a time showed that removing components did not result in an increase in Cronbach’s alpha. Thus, each component of FNLQ show an acceptable internal consistency with the overall questionnaire

### 3.4. Construct Validity

Only 16 components of the practice dimensions were analyzed by EFA and CFA. For the EFA part, the KMO was 0.923, showing sampling adequacy, and Bartlett’s test confirmed that the factor analysis was appropriate (*p* < 0.001). Three factors were extracted with eigenvalues greater than 1, and the cumulative contribution of variance accounted for 60.86% of the overall variance. CFA indicators of the practice domain showed an acceptable fit in general. The RMSEA was 0.048 (≤0.08), and the χ^2^/df were 2.915 less than 3.0. The value of GFI and AGFI were 0.891 and 0.876, respectively.

### 3.5. Content Validity

As shown in Table 3, the Pearson correlation coefficients among different dimensions ranged from 0.38~0.89. The correlation coefficients between each dimension and the overall questionnaire ranged from 0.665 to 0.887, especially the coefficients of dimensions of knowledge and understanding, selecting food, preparing food, and eating, which were more than 0.6 and showed a strong correlation with the overall questionnaire. The Pearson correlation coefficients between each component and the overall questionnaire ranged from 0.201 to 0.779. Only one coefficient of component was less than 0.3, named “Understanding that a rational diet is an important basis for maintaining health and avoiding disease”.

### 3.6. Assessing Food and Nutrition Literacy and Its Related Factors

As shown in Table 4, the average FNLQ score of all participants was 64.08 ± 12.77, and participants’ scores ranged from 12 to 95. Among the dimensions, the score for knowledge (73.07 ± 16.46) was higher than the score for practice dimensions. The scores of electing food, preparing food, and eating were 55.11 ± 16.63, 58.69 ± 19.33, and 65.44 ± 11.09, respectively. The score for selecting food was the lowest (55.11 ± 16.63). In addition, 80 was setting as the nutritional literacy threshold, only 12.2% met it in this survey.

The participants who were female, with higher education levels, stable marital status (never married or married), healthcare-related education or work experience (YES), or suffering from chronic disease would have significantly higher food and nutrition literacy (R^2^ = 0.287, F = 244.132, *p* < 0.01), as shown in Table 5. Interestingly, there were no uniform results on the relationship between family income and nutrition literacy scores. Logistic regression was also performed for analyzing the Food and Nutrition Literacy related factors among excellent scores (≥80) (shown in Table S1). Similarly, excellent scores were more likely to be female, well-educated, have a stable marital status (never married or married), healthcare-related education or work experience (YES), and not be suffering from chronic disease. 

## 4. Discussion

To our knowledge, this is the first reported FNLQ for adults in China. The questionnaire including 20 core components, distributing in four dimensions of one knowledge and three practices. The overall FNLQ is a tool with acceptable reliability and validity, which can be used to evaluate the food and nutrition literacy of adults in China.

Food is the carrier of nutrition. It has been revealed that nutrition literacy and food literacy are specific forms of health literacy and represent distinct but complementary concepts [23]. The NLAI for American adults includes serial points: knowledge, understanding the relationships between nutrition and health, as well as skills such as classification and measurement of foods, numeracy, and label reading [20,27]. The Food Literacy of Australian contained planning and management, selection, preparation, and eating domains [28]. There is an integrated Food Literacy tool for Belgium, whose definition of Food and Nutrition Literacy is as following: “Food literacy is the interrelated combination of knowledge, skills, and self-efficacy on food planning, selecting foods, and food preparation, eating and evaluating information about food with the ultimate goal of developing a lifelong healthy, sustainable and gastronomic relationship with food” [28]. Beyond the ability to access and understand nutrition information, we also paid attention on the ability to communicate and act upon this information [24]. As our previous study, the term Food and Nutrition Literacy was defined as a collection of interrelated knowledge, and practices required to plan, manage, select, prepare, and eat foods to meet requirements and determine food intake [9]. The FNLQ was developed based on the conceptual framework using a literature review [22,24,27,28,29,30,31], expert interviews, and qualitative consensus study, which included four dimensions of food and nutrition knowledge: selection, preparation of food and eating and three levels of functional, interactive, and critical literacy. The study produced I-CVI scores ranging from 0.83 to 1.0, with S-CVI scores of 0.98, 0.93, 1.00, and 0.93 on the food and nutrition knowledge and understanding, ability of access and selecting food, preparing food, and eating, respectively. According to the results of validity test, the components were deemed relevant in measuring the knowledge, and practice of an individual regarding food nutrition literacy. 

The total Cronbach’s α was 0.858, which indicated that the overall questionnaire had acceptable internal consistency. The structural validity of the scale refers to the degree of consistency between the actual measurement and the theoretical conception model [32]. For the practice domain, EFA extracted three factors that were included in the conceptual framework but in a slightly different model. The Pearson correlation coefficients between the four dimensions and the overall questionnaire were more than 0.6, which indicated a strong correlation. In addition, the result of CFA showed an acceptable fit in general. The results showed that χ2/DF was 2.915 (<3.0), indicating that the adaptation was ideal; RMSEA was 0.048 (<0.08), indicating that the adaptation was ideal; the value of GFI and AGFI were 0.891 and 0.876, respectively, indicating that the adaptation was acceptable. All the above indicate that the structural equation model of the FNLQ is successfully established, and the actual measurement basically aligns with the theoretical simulation.

Using the FNLQ, we assessed the Food and Nutrition Literacy level of 8510 adults. The average FNLQ score of all participants was (64.08 ± 12.77), and the score for the knowledge and understanding dimension was higher than that for the practice dimensions. the understanding and judgment of “good dietary patterns are the foundation of adequate nutrition” were poor with low scores of 0.785 ± 0.60; “whole grains belong to grains and tubers” were good with high scores of 1.722 ± 0.385. The core component “Choosing a healthy diet and enjoy your food” was good with 3.338 ± 0.774 (full marks = 4), whereas “understanding that a rational diet is an important basis for maintaining health and avoiding disease” was poor with 0.785 ± 0.603. In terms of practice, the score for selecting food was the lowest (55.11 ± 16.63), and the components of “What do you value most when you buy qualified raw meat in the supermarket?”, “If you eat out, above which the sanitary level of restaurant will you choose?”, and “the nutritional labels” were the lowest 3 with score 0.175 ± 0.565 (only 8.8% correct rate), 0.608 ± 0.920, and 0.994 ± 0.586, respectively. The core component “Making your own food, eating out less and sharing meals with family” was good with 3.377 ± 0.772 (full marks = 4), whereas “Being able to read and understand food nutrition labels” was poor with 2.239 ± 1.27 (full marks = 6). In the preparing food part, “What is your attitude to ‘Tropical fruits (such as bananas) can be stored in refrigerators’?” were lowest with a score of 0.935 ± 0.623. The core component “Being able to estimate food portion size” was good with 2.674 ± 1.193 (full marks = 2), whereas “Being able to match food rationally” was poor with 0.983 ± 1.000 (full marks = 2). In the eating part, the score of “How many glasses of water did you drink per day during the past 7 days? (1 cup = 200 mL)” and “How often do you drink alcohol?” were lowest (0.384 ± 0.511 and 0.587 ± 0.570, respectively). The core component “Preparing meals on demand, eating in a civilized manner, and eliminating waste” was good with 3.414 ± 0.743 (full marks = 4), whereas “Balance eating and movement, measure and evaluate your weight regularly” was poor with 6.097 ± 1.583 (full marks = 10). This suggests that we need to pay greater attention to the weaker link, which is the development of practical food and nutrition practices in China.

The overall dietary quality index was used to determine the optimal Food and Nutrition Literacy cutoff score. However, the cut points of Food and Nutrition Literacy could not be identified as the results of no dietary intake data in this study. If the score of 80 was set as the nutritional literacy threshold, only 12.2% of the participants met it in this survey. However, 90% and above participants came from the first-tier cities in China, such as Beijing, Shanghai, Guangzhou, and Shenzhen. The level of food and nutrition literacy is still too low, which should be given more attention. In Turkey, the Nutrition Literacy of young adults has been evaluated, and the scores acquired by females were significantly higher than males [33]. In an Indian investigation, the related factors associated with low nutrient intake or/and unhealthy eating habits were living in rural areas, youth, and low education [34]. The results of a study in Hong Kong showed that nutritional labeling literacy levels are quite low, especially among those with less education and/or older age [35]. Likewise, among Portuguese adults, participants with higher education, following a specific diet, having an adequate BMI, having family members trained in the field of nutrition, and those studying or working in the health sciences reported higher levels of Nutrient nutrition [36]. Consistent results were found in our study, and it has been shown that Food and Nutrition Literacy levels was were with age, higher education, stable marital status (never married or married), healthcare-related education or work experience (YES), and proper BMI and health status (without suffering the chronic disease). Interestingly, there were no uniform results on the relationship between family income and nutrition literacy scores. It was speculated that the family economic income is related to the regional economy of the sample, which also provides a new sociological perspective for future nutrition literacy research. In fact, BMI data are collected as continuous variables. When we considered that when BMI, analyzed as a continuous variable, did not affect the score of literacy significantly, on the contrary, there will be significant differences between appropriate (18.5~23.9) and abnormal (<18.5 or ≥24.0) BMI, and the explanation of influencing factors will also be more valuable. However, there were only 31.7% participants with abnormal (<18.5 or ≥24.0) BMI in this study, which was lower than the 38.5% found in the data from the Report on the Nutrition and Chronic Disease Status of Chinese Residents (2020). From the perspective of BMI, the sample used in this study is not representative enough to explain the national situation in China. Therefore, further research is needed to explore the relationship between BMI and Food and Nutrition Literacy.

The limitations of this study are listed as follows: (1) As the main body of labor resources, the food environments of work units and families are an important factor affecting individual eating behavior and nutritional health status. However, no consideration was given to the food environment in the present study. (2) When issuing the questionnaire, there was the choice of convenience sampling (for example, no rural data) and the risk of self-reported bias. (3) Some indicators in the process of questionnaire verification may only reach an acceptable level and can be improved in subsequent research. Hence, the effectiveness, acceptability, and universal applicability of the FNLQ still needs to be the future verified. The development and validation of an appropriate instrument is an essential step for FNLQ research.

## 5. Conclusions

Taken together, the FNLQ has good reliability to some extent, and it could potentially be a useful instrument for assessing Food and Nutrition Literacy. Of course, because the investigation sites and sample could not represent Chinese adults, a nationwide survey of Food and Nutrition Literacy was necessary to identify the target population for further nutrition education to develop targeted interventions to improve Food and Nutrition Literacy and dietary quality, thus further improving their health.

## Figures and Tables

**Table 1:** The core components of food and nutrition literacy.

Domain	Dimension	Components	The Second Round Consultation				
			N ^#^	I-CVI	Pc	K *	S-CVI
Knowledge	Food and nutrition Knowledge	Understanding that a healthy diet should be followed at every stage of life. ^a^	12	1.00	0.0002	1.00	0.98
		2. Understanding that a rational diet is an important basis for maintaining health and avoiding disease. ^a^	12	1.00	0.0002	1.00	
		3. Knowing about food classification, sources, and main nutritional characteristics. ^a^	12	1.00	0.0002	1.00	
		4. Choosing a healthy diet and enjoy your food. ^a^	11	0.92	0.0029	0.92	
Practices	Access to and planning and selecting for food	5. Making your own food, eating out less and sharing meals with family. ^a^	10	0.83	0.0161	0.83	0.93
		6. Being able to choose safe and hygienic food stores and restaurants. ^a^	10	0.83	0.0161	0.83	
		7. Being able to judge food quality and to choose fresh and healthy food. ^a^	11	0.92	0.0029	0.92	
		8. Being able to read and understand food nutrition labels. ^a^	12	1.00	0.0002	1.00	
		9. Paying attention to nutrition and health information, identifying, and applying the right information. ^c^	12	1.00	0.0002	1.00	
		10. Being able to choose healthy food and fortified food correctly. ^a^	12	1.00	0.0002	1.00	
	Preparing and marking food	11. Being able to estimate food portion size. ^a^	12	1.00	0.0002	1.00	1.00
		12. Being able to match food rationally. ^a^	12	1.00	0.0002	1.00	
		13. Being able to store, prepare, process, and cook food in an appropriate manner. ^a^	12	1.00	0.0002	1.00	
	Eating	14. Eating regular meals and having a good breakfast. ^a^	12	1.00	0.0002	1.00	0.93
		15. Eating a variety of foods, mainly grains, eating more fruits and vegetables, and drinking plenty of water. ^a^	11	0.92	0.0029	0.92	
		16. Eating appropriate amount of fish, poultry, eggs, lean meat, and adequate milk and beans. ^a^	12	1.00	0.0002	1.00	
		17. Eating less salt and less oil, controlling sugar, and limiting wine. ^a^	11	0.92	0.0029	0.92	
		18. Preparing meals on demand, eating in a civilized manner, and eliminating waste. ^a^	10	0.83	0.0161	0.83	
		19. Respecting different food cultures and paying attention to table manners. ^b^	10	0.83	0.0161	0.83	
		20. Balance eating and movement, measure and evaluate your weight regularly. ^a^	12	1.00	0.0002	1.00	

**^#^**: total number of experts who scored 3 or 4; K *: value of kappa; ^a^ Functional literacy; ^b^ Interactive literacy; ^c^ Critical literacy. I-CVI, Item-Level Content Validity Index; S-CVI, Scale-Level Content Validity Index.

**Table 2:** Demographic characteristics of participants, *n* (%).

Characteristics	Total (*N* = 8510)	Reliability and Validity Tests
		(*N* = 841)
Age (mean ± SD)	34.47 ± 7.41	33.88 ± 7.38
BMI (mean ± SD)	21.57 ± 2.92	21.37 ± 2.80
Gender		
Male (*n*, %)	4577 (53.8%)	438 (52.1%)
Female (*n*, %)	3933 (46.2%)	403 (47.9%)
Education level		
Junior high school degree or below	382 (4.5%)	40 (4.8%)
Senior high school degree	2986 (35.1%)	285 (33.9%)
Bachelor’s/Technical degree or above	5142 (60.4%)	516 (61.4%)
Marriage status		
Never married	1406 (16.5%)	150 (17.8%)
Married	6276 (73.7%)	614 (73.0%)
Divorced	765 (9%)	69 (8.2%)
Other	63 (0.7%)	8 (1.0%)
Healthcare related work experience		
Yes	4395 (51.6%)	441 (52.4%)
No	4115 (48.4%)	400 (47.6%)
Family income *		
≤5000 CNY/month	784 (9.2%)	66 (7.8%)
5000~8000 CNY/month	1461 (17.2%)	156 (18.5%)
8000~13,000 CNY/month	2429 (28.5%)	232 (27.6%)
13,000~17,000 CNY/month	1752 (20.6%)	170 (20.2%)
17,000~24,000 CNY/month	1241 (14.6%)	119 (14.1%)
>24,000 CNY/month	843 (9.9%)	98 (11.7%)
Chronic diseases		
None	5331 (62.6%)	540 (64.2%)
Single disease	811 (9.5%)	70 (8.3%)
Multimorbidity ^&^	2368 (27.8%)	231 (27.5%)

Note: The sum of percentages did not add up to 100.00% because of the default value. * Exchange rate of CNY to USD is about RMB 640 equal to USD 100. ^&^ Suffered from the two diseases at the same time were judged as multimorbidity, including dyslipidemia, diabetes or elevated blood sugar, hypertension, cancer and other malignant tumors, chronic lung diseases such as bronchitis, emphysema, pulmonary heart disease, liver diseases, heart disease, stroke, kidney disease, stomach disease or digestive system disease, emotional and mental problems, etc.

**Table 3:** Pearson correlation coefficient among dimensions of FNLQ (*n* = 841, mean ± SD).

Dimensions	Knowledge (8′)	Selecting Food (30′)	Preparing Food (22′)	Eating (40′)	Total (100′)
Knowledge	--	0.439 **	0.375 **	0.668 **	0.665 **
Selecting food	0.439 **	--	0.797 **	0.480 **	0.887 **
Preparing food	0.375 **	0.797 **	--	0.391 **	0.834 **
Eating	0.668 **	0.480 **	0.391 **	--	0.782 **

Note: --: no data; **: *p* < 0.01.

**Table 4:** Distribution of food and nutrition literacy in Chinese adults (*n* = 8510, mean ± SD).

Variables	Total (100′)	Knowledge (8′)	Selecting Food (30′)	Preparing Food (22′)	Eating (40′)
Total	64.08 ± 12.77	5.84 ± 1.32	16.53 ± 4.99	12.91 ± 4.25	28.79 ± 4.89
Age					
15~30	63.93 ± 12.31	5.87 ± 1.31	16.59 ± 4.93	12.79 ± 4.07	28.68 ± 4.83
31~45	64.26 ± 12.89	5.83 ± 1.29	16.55 ± 5.02	12.99 ± 4.29	28.89 ± 4.90
46~60	63.18 ± 13.39	5.85 ± 1.52	16.18 ± 4.96	12.70 ± 4.53	28.45 ± 4.96
Gender					
Male	61.56 ± 12.44	5.68 ± 1.35	15.57 ± 4.80	11.99 ± 4.03	28.30 ± 5.07
Female	67.02 ± 12.50 ^a^	6.03 ± 1.26 ^a^	17.65 ± 4.98 ^a^	13.98 ± 4.26 ^a^	29.36 ± 4.59 ^a^
Education level					
Junior high school degree	52.63 ± 10.65	4.85 ± 1.67	12.45 ± 3.45	9.59 ± 2.83	25.74 ± 5.98
Senior high school degree	59.27 ± 12.18 ^a^	5.49 ± 1.46 ^a^	14.61 ± 4.48 ^a^	11.40 ± 3.92 ^a^	27.77 ± 5.32 ^a^
Bachelor’s/Technical degree or above	67.72 ± 11.79 ^a,b^	6.13 ± 1.10 ^a,b^	17.95 ± 4.83 ^a,b^	14.04 ± 4.13 ^a,b^	29.61 ± 4.30 ^a,b^
Marriage status					
Never married	65.96 ± 11.58	6.11 ± 1.25	17.35 ± 4.79	13.58 ± 4.01	28.92 ± 4.54
Married	65.09 ± 12.62 ^a^	5.90 ± 1.23 ^a^	16.87 ± 4.98 ^a^	13.21 ± 4.26 ^a^	29.11 ± 4.71 ^a^
Divorced	53.20 ± 10.17 ^a^	4.99 ± 1.63 ^a,b^	12.50 ± 3.24 ^a,b^	9.46 ± 2.72 ^a,b^	26.25 ± 5.84 ^a,b^
Other	53.26 ± 13.64 ^b,c^	4.58 ± 1.85 ^a,b^	13.25 ± 4.50 ^a,b^	10.29 ± 3.67 ^a,b^	25.14 ± 6.13 ^a,b^
Healthcare related work experience					
Yes	60.91 ± 12.56	5.68 ± 1.39	15.22 ± 4.81	11.66 ± 3.97	28.35 ± 5.18
No	67.46 ± 12.11 ^a^	6.02 ± 1.21 ^a^	17.93 ± 4.79 ^a^	14.25 ± 4.13 ^a^	29.26 ± 4.51 ^a^
Family income *					
≤5000 CNY/month	66.79 ± 10.65	6.27 ± 1.28	17.62 ± 4.35	14.10 ± 3.76	28.80 ± 4.30
5000~8000 CNY/month	65.30 ± 12.44 ^a^	5.90 ± 1.25 ^a^	16.90 ± 4.93 ^a^	13.60 ± 4.23 ^a^	28.91 ± 4.68
8000~13,000 CNY/month	61.06 ± 12.43 ^a,b^	5.65 ± 1.32 ^a,b^	15.32 ± 4.79 ^a,b^	11.85 ± 4.09 ^a,b^	28.24 ± 4.97 ^a,b^
13,000~17,000 CNY/month	61.82 ± 12.40 ^a,b^	5.68 ± 1.32 ^a,b^	15.65 ± 4.81 ^a,b^	11.99 ± 4.02 ^a,b^	28.50 ± 4.99 ^b^
17,000~24,000 CNY/month	65.44 ± 13.36 ^a,b,c,d^	5.94 ± 1.41 ^a,b^	17.18 ± 5.09 ^a,c,d^	13.36 ± 4.36 ^a,b,d^	28.95 ± 5.04 ^b^
>24,000 CNY/month	70.83 ± 12.31 ^a,c,d,e^	6.14 ± 1.15 ^a,c,d,e^	19.26 ± 4.95 ^a,c,d,e^	14.93 ± 4.16 ^a,c,d,e^	30.50 ± 4.67 ^a,b,c,d,e^
Chronic diseases					
None	67.10 ± 12.51	6.02 ± 1.27	17.77 ± 4.94	13.99 ± 4.18	29.32 ± 4.71
Single disease	64.21 ± 13.21 ^a,c^	5.79 ± 1.43 ^a,c^	16.87 ± 4.85 ^a,c^	13.54 ± 4.17 ^a,c^	28.01 ± 5.09 ^a,c^
Multimorbidity ^&^	57.22 ± 10.30 ^a,b^	5.74 ± 1.30 ^a,b^	13.63 ± 3.83 ^a,b^	10.26 ± 3.14 ^a,b^	27.87 ± 5.04 ^a^
BMI					
18.5~23.9	64.71 ± 12.70	5.88 ± 1.27	16.73 ± 4.99	13.06 ± 4.25	29.03 ± 4.85
<18.5 or ≥24.0	62.74 ± 12.79 ^a^	5.76 ± 1.40 ^a^	16.10 ± 4.95 ^a^	12.59 ± 4.23 ^a^	28.28 ± 4.92 ^a^

Note: Different superscript characters (a, b, c, d, e) indicate significant differences among groups (*p* < 0.05). * Exchange rate of CNY to USD is about RMB 640 equal to USD 100. ^&^ Suffered from the two diseases at the same time were judged as multimorbidity, including dyslipidemia, diabetes or elevated blood sugar, hypertension, cancer and other malignant tumors, chronic lung diseases such as bronchitis, emphysema, pulmonary heart disease, liver diseases, heart disease, stroke, kidney disease, stomach disease or digestive system disease, emotional and mental problems, etc.

**Table 5:** Multiple linear regression analysis of food and nutrition-literacy-related factors among Chinese adults (*n* = 8510).

Variables *	β	SE	B	*T*	*p*
(Constant)	56.09	0.75		74.94	0.00
Gender (Female)	3.16	0.24	0.12	13.15	0.00
Education level					
Junior high school degree or below	--				
Senior high school degree	4.18	0.59	0.16	7.06	0.00
Bachelor’s/Technical degree or above	9.70	0.59	0.37	16.38	0.00
Marriage status					
Never married	--				
Married	0.90	0.32	0.03	2.77	0.01
Divorced	−5.35	0.51	−0.12	−10.41	0.00
Other	−7.27	1.40	−0.05	−5.20	0.00
Healthcare related work experience (YES)	3.34	0.25	0.13	13.54	0.00
Family income					
≤5000 RMB/month	--				
5000~8000 RMB/month	−0.01	0.48	−0.00	−0.03	0.98
8000~13,000 RMB/month	−2.87	0.45	−0.10	−6.34	0.00
13,000~17,000 RMB/month	−1.79	0.47	−0.06	−3.77	0.00
17,000~24,000 RMB/month	−0.06	0.50	−0.00	−0.11	0.91
>24,000 RMB/month	3.89	0.54	0.09	7.15	0.00
Chronic diseases					
None	--				
Single disease	−2.34	0.41	−0.05	−5.74	0.00
Multimorbidity ^&^	−5.40	0.29	−0.19	−18.70	0.00

Variable values: Sex (Male = 0, Female = 1); Healthcare related education or work experience (No = 0, Yes = 1). --: no data; *: Exchange rate of CNY to USD is about RMB 640 equal to USD 100. ^&^: Suffered from the two diseases at the same time were judged as multimorbidity, including dyslipidemia, diabetes or elevated blood sugar, hypertension, cancer and other malignant tumors, chronic lung diseases such as bronchitis, emphysema, pulmonary heart disease, liver diseases, heart disease, stroke, kidney disease, stomach disease or digestive system disease, emotional and mental problems, etc.

## Supplementary Materials

The following supporting information can be downloaded at: https://www.mdpi.com/article/10.3390/nu14091933/s1. Table S1: Food and nutrition literacy questionnaire (English version).
