# Supplementary material for: Development and Validation of a Food and Nutrition Literacy Questionnaire for Chinese Adults
Source: Nutrients. 2022 May 5;14(9):1933. doi: 10.3390/nu14091933 (PMC9104569; doi:10.3390/nu14091933)
Supplement: Supplementary file 1 [file nutrients-14-01933-s001.zip › nutrients-1691962-supplementary.pdf]

Table S1. Logistic regression analysis of food and nutrition literacy-related factors among Chinese adults with excellent score (≥80) (n = 8510).

**Variables**	**B**	**SE**	**Wald**	***P***	**OR (OR95%CI)**
(Constant)	−2.252	0.631	12.738	0.000	
Gender (Male)	−0.551	0.072	58.804	0.000	0.576 (0.500~0.663)
Education level					
Junior high school degree or below	−1.923	0.458	17.655	0.000	0.146(0.060~0.358)
Senior high school degree	−0.781	0.092	72.325	0.000	0.458(0.382~0.548)
Bachelor’s/Technical degree or above	--				
Marriage status					
Never married	0.259	0.619	0.175	0.675	1.296(0.385~4.356)
Married	0.606	0.614	0.974	0.324	1.832(0.551~6.099)
Divorced	−1.297	0.709	3.347	0.067	0.273(0.068~1.097)
Other	--				
Healthcare related work experience (NO)	-0.391	0.073	28.434	0.000	0.676(0.586~0.781)
Family income					
≤5000 RMB/month	−1.267	0.154	68.017	0.000	0.282(0.208~0.381)
5000 ~ 8000 RMB/month	−0.722	0.117	37.799	0.000	0.486(0.386~0.611)
8000 ~ 13,000 RMB/month	−1.020	0.113	81.900	0.000	0.361(0.289~0.450)
13,000 ~ 17,000 RMB/month	−0.947	0.121	61.484	0.000	0.388(0.306~0.492)
17,000 ~ 24,000 RMB/month	−0.489	0.116	17.705	0.000	0.613(0.488~0.770)
>24,000 RMB/month	--				
Chronic diseases					
None	1.491	0.135	121.342	0.000	4.443(3.407~5.793)
Single disease	0.956	0.175	29.842	0.000	2.602(1.847~3.668)
Multimorbidity *	--				

Note: Variable values: Sex (Male = 0, Female = 1); Healthcare related education or work experience (No = 0, Yes =1). * Exchange rate of CNY to USD is about 640 RMB yuan equal to 100 US dollars. & Suffered from the two diseases at the same time were judged as multi-morbidity, including dyslipidemia, diabetes or elevated blood sugar, hypertension, cancer and other malignant tumors, chronic lung diseases such as bronchitis, emphysema, pulmonary heart disease, liver diseases, heart disease, stroke, kidney disease, stomach disease or digestive system disease, emotional and mental problems, etc.
